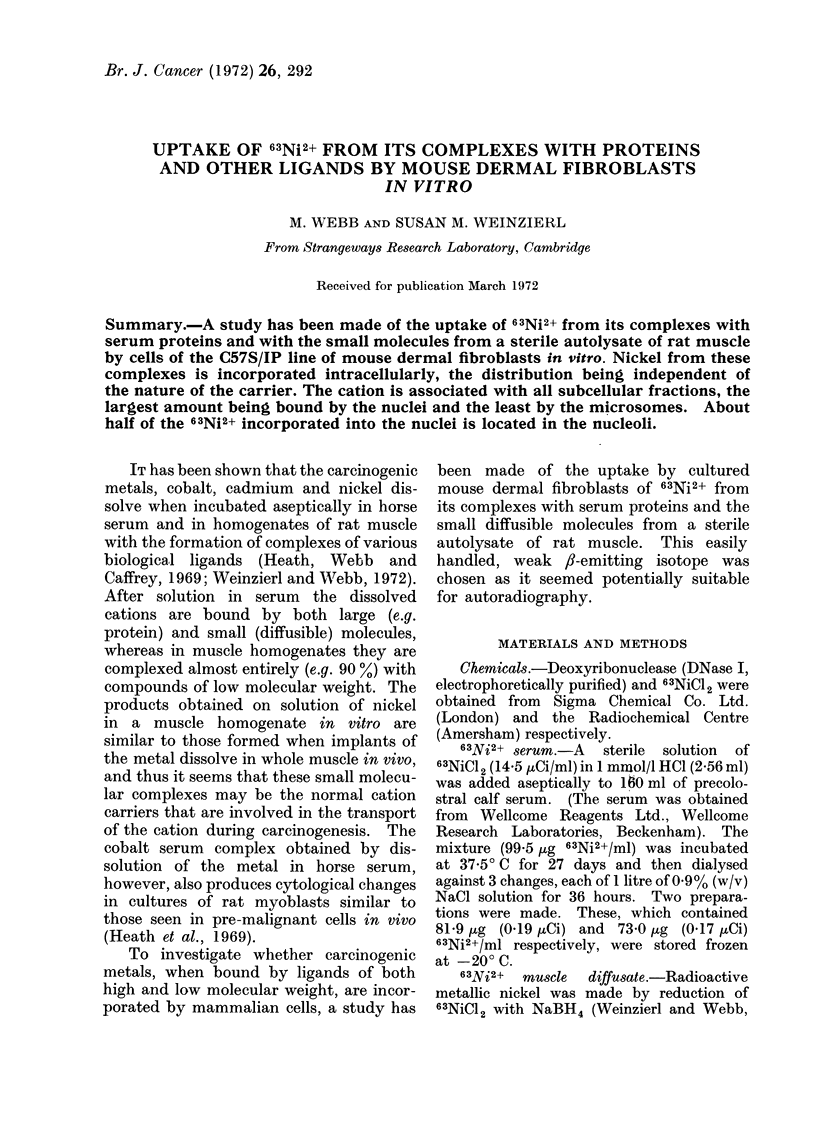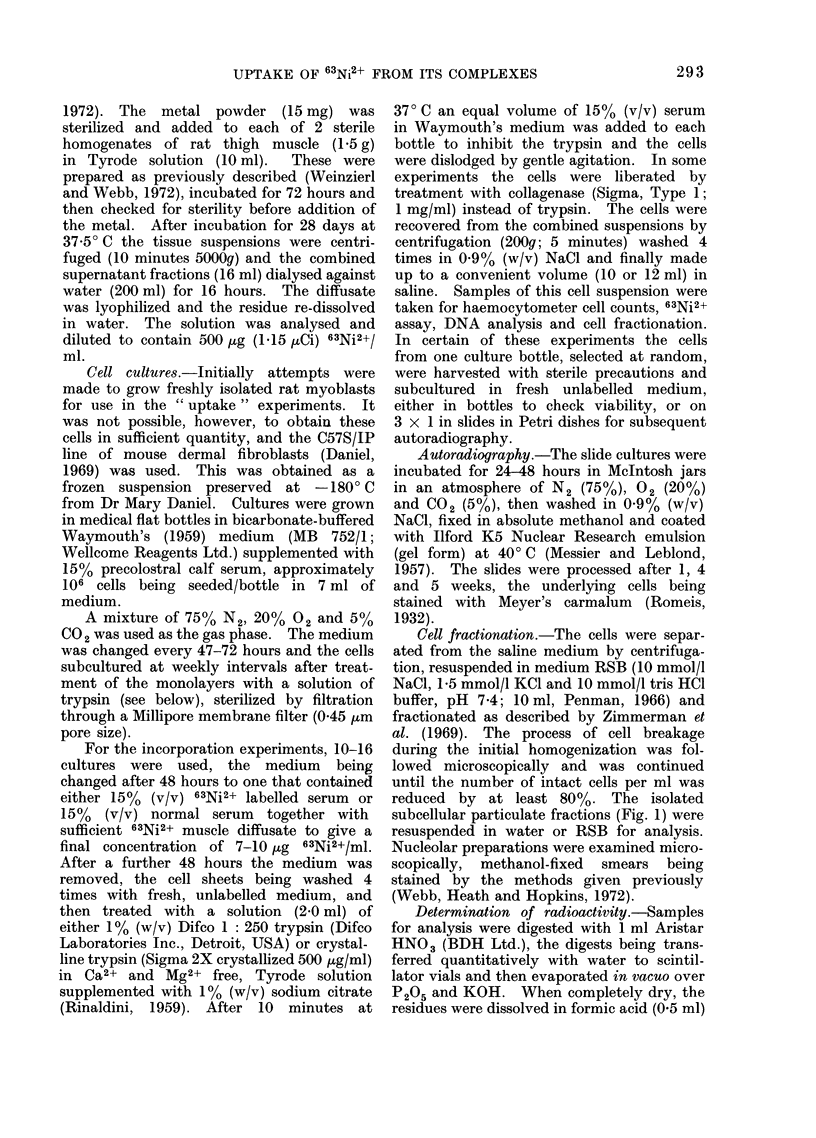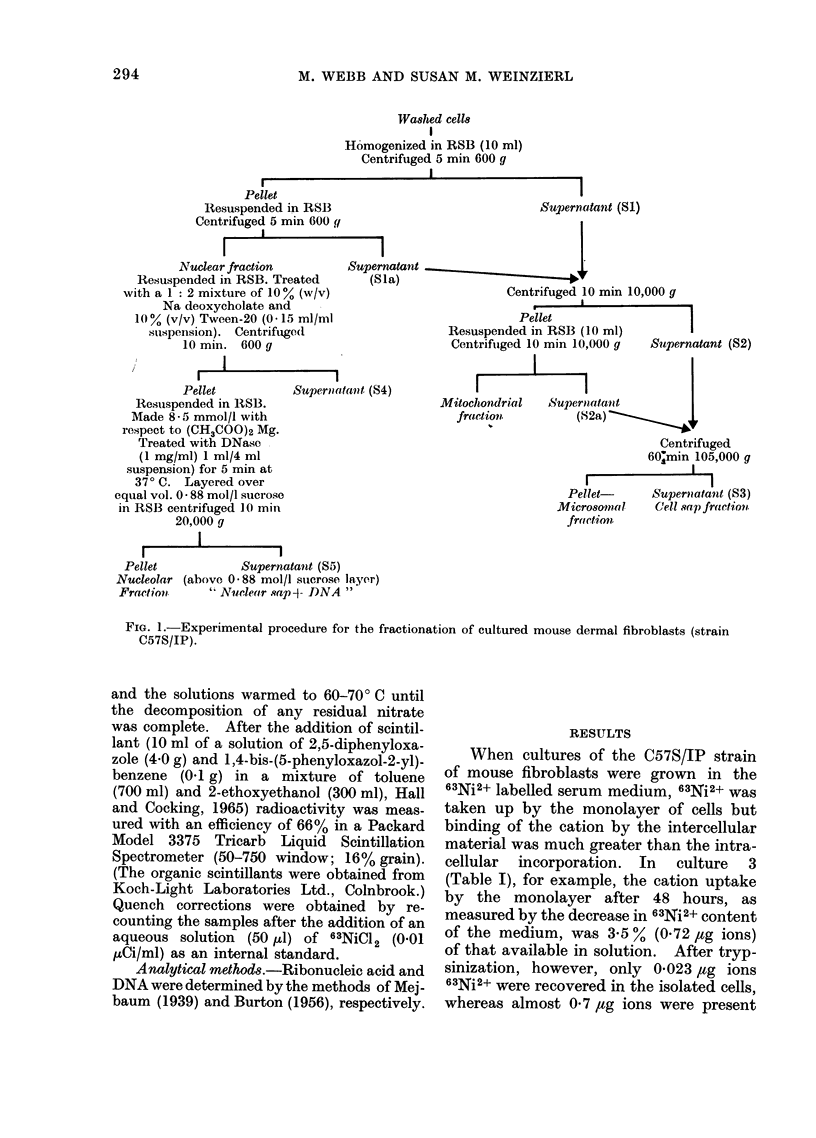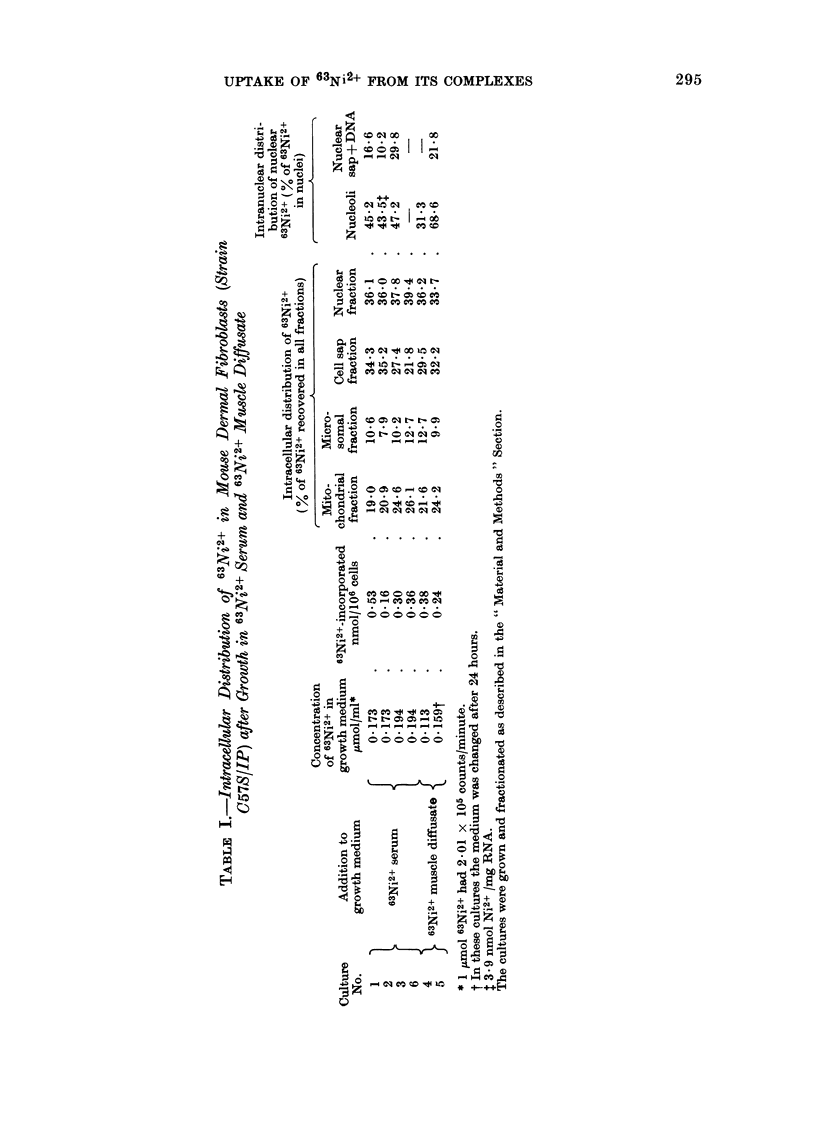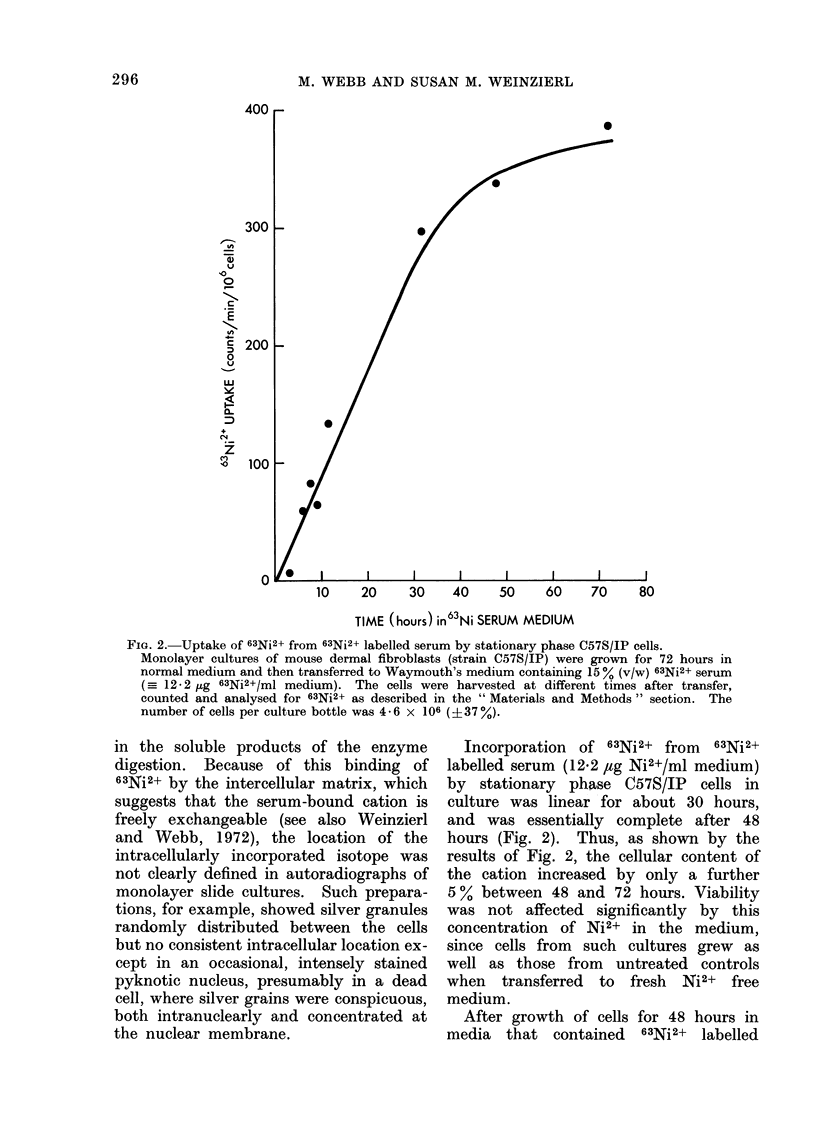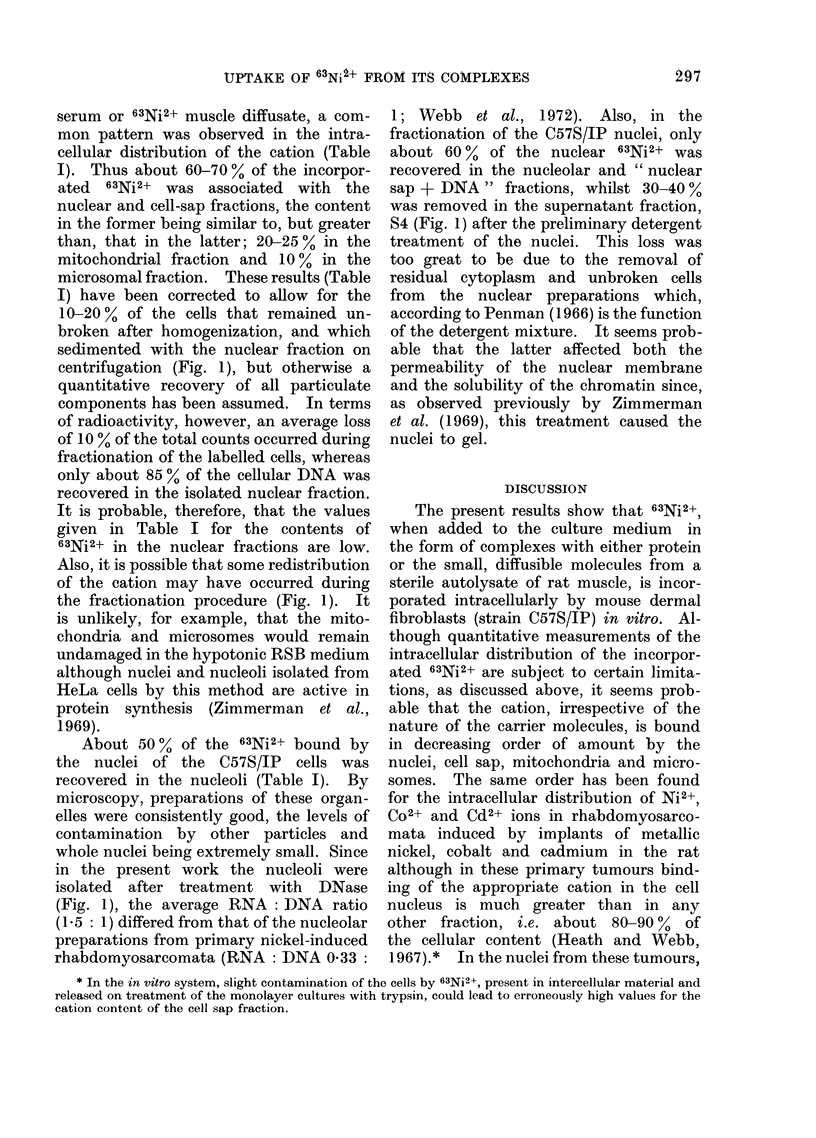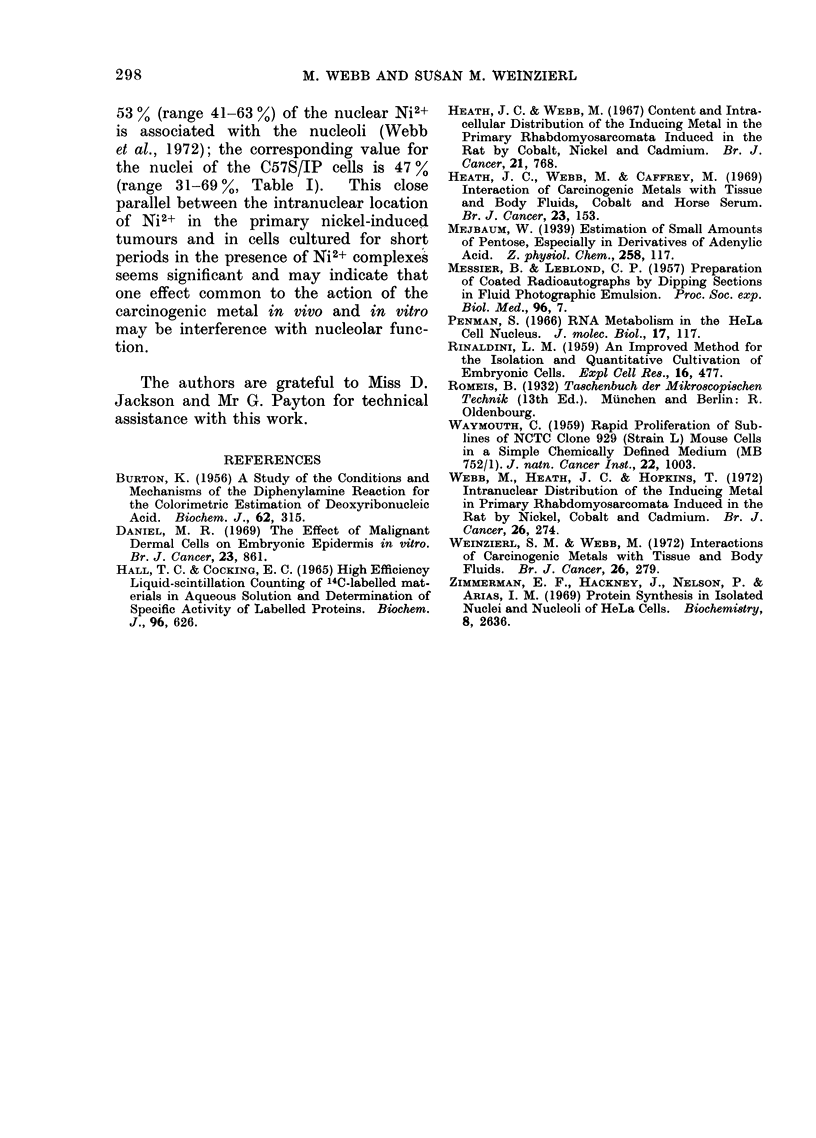# Uptake of ^63^Ni^2+^ from its Complexes with Proteins and other Ligands by Mouse Dermal Fibroblasts in vitro

**DOI:** 10.1038/bjc.1972.39

**Published:** 1972-08

**Authors:** M. Webb, Susan M. Weinzierl

## Abstract

A study has been made of the uptake of ^63^Ni^2+^ from its complexes with serum proteins and with the small molecules from a sterile autolysate of rat muscle by cells of the C57S/IP line of mouse dermal fibroblasts *in vitro.* Nickel from these complexes is incorporated intracellularly, the distribution being independent of the nature of the carrier. The cation is associated with all subcellular fractions, the largest amount being bound by the nuclei and the least by the microsomes. About half of the ^63^Ni^2+^ incorporated into the nuclei is located in the nucleoli.


*Br. J. Cancer* (1972) **26**, 292

### UPTAKE OF $^{63}Ni^{2+}$ FROM ITS COMPLEXES WITH PROTEINS AND OTHER LIGANDS BY MOUSE DERMAL FIBROBLASTS *IN VITRO*

M. WEBB AND SUSAN M. WEINZIERL

*From Strangeways Research Laboratory, Cambridge*

Received for publication March 1972

**Summary.—A study has been made of the uptake of $^{63}Ni^{2+}$ from its complexes with serum proteins and with the small molecules from a sterile autolysate of rat muscle by cells of the C57S/IP line of mouse dermal fibroblasts *in vitro*. Nickel from these complexes is incorporated intracellularly, the distribution being independent of the nature of the carrier. The cation is associated with all subcellular fractions, the largest amount being bound by the nuclei and the least by the microsomes. About half of the $^{63}Ni^{2+}$ incorporated into the nuclei is located in the nucleoli.**

IT has been shown that the carcinogenic metals, cobalt, cadmium and nickel dissolve when incubated aseptically in horse serum and in homogenates of rat muscle with the formation of complexes of various biological ligands (Heath, Webb and Caffrey, 1969; Weinzierl and Webb, 1972). After solution in serum the dissolved cations are bound by both large (*e.g.* protein) and small (diffusible) molecules, whereas in muscle homogenates they are complexed almost entirely (*e.g.* 90 %) with compounds of low molecular weight. The products obtained on solution of nickel in a muscle homogenate *in vitro* are similar to those formed when implants of the metal dissolve in whole muscle *in vivo*, and thus it seems that these small molecular complexes may be the normal cation carriers that are involved in the transport of the cation during carcinogenesis. The cobalt serum complex obtained by dissolution of the metal in horse serum, however, also produces cytological changes in cultures of rat myoblasts similar to those seen in pre-malignant cells *in vivo* (Heath *et al.*, 1969).

To investigate whether carcinogenic metals, when bound by ligands of both high and low molecular weight, are incorporated by mammalian cells, a study has been made of the uptake by cultured mouse dermal fibroblasts of $^{63}Ni^{2+}$ from its complexes with serum proteins and the small diffusible molecules from a sterile autolysate of rat muscle. This easily handled, weak $\beta$-emitting isotope was chosen as it seemed potentially suitable for autoradiography.

### MATERIALS AND METHODS

*Chemicals.*—Deoxyribonuclease (DNase I, electrophoretically purified) and $^{63}NiCl_2$ were obtained from Sigma Chemical Co. Ltd. (London) and the Radiochemical Centre (Amersham) respectively.

*$^{63}Ni^{2+}$ serum.*—A sterile solution of $^{63}NiCl_2$ (14·5 $\mu$Ci/ml) in 1 mmol/l HCl (2·56 ml) was added aseptically to 160 ml of precolostral calf serum. (The serum was obtained from Wellcome Reagents Ltd., Wellcome Research Laboratories, Beckenham). The mixture (99·5 $\mu$g $^{63}Ni^{2+}$/ml) was incubated at 37·5° C for 27 days and then dialysed against 3 changes, each of 1 litre of 0·9% (w/v) NaCl solution for 36 hours. Two preparations were made. These, which contained 81·9 $\mu$g (0·19 $\mu$Ci) and 73·0 $\mu$g (0·17 $\mu$Ci) $^{63}Ni^{2+}$/ml respectively, were stored frozen at −20° C.

*$^{63}Ni^{2+}$ muscle diffusate.*—Radioactive metallic nickel was made by reduction of $^{63}NiCl_2$ with $NaBH_4$ (Weinzierl and Webb,

1972). The metal powder (15 mg) was sterilized and added to each of 2 sterile homogenates of rat thigh muscle (1·5 g) in Tyrode solution (10 ml). These were prepared as previously described (Weinzierl and Webb, 1972), incubated for 72 hours and then checked for sterility before addition of the metal. After incubation for 28 days at 37·5° C the tissue suspensions were centrifuged (10 minutes 5000*g*) and the combined supernatant fractions (16 ml) dialysed against water (200 ml) for 16 hours. The diffusate was lyophilized and the residue re-dissolved in water. The solution was analysed and diluted to contain 500 $\mu$g (1·15 $\mu$Ci) $^{63}Ni^{2+}$/ml.

*Cell cultures.*—Initially attempts were made to grow freshly isolated rat myoblasts for use in the "uptake" experiments. It was not possible, however, to obtain these cells in sufficient quantity, and the C57S/IP line of mouse dermal fibroblasts (Daniel, 1969) was used. This was obtained as a frozen suspension preserved at −180° C from Dr Mary Daniel. Cultures were grown in medical flat bottles in bicarbonate-buffered Waymouth's (1959) medium (MB 752/1; Wellcome Reagents Ltd.) supplemented with 15% precolostral calf serum, approximately $10^6$ cells being seeded/bottle in 7 ml of medium.

A mixture of 75% $N_2$, 20% $O_2$ and 5% $CO_2$ was used as the gas phase. The medium was changed every 47–72 hours and the cells subcultured at weekly intervals after treatment of the monolayers with a solution of trypsin (see below), sterilized by filtration through a Millipore membrane filter (0·45 $\mu$m pore size).

For the incorporation experiments, 10–16 cultures were used, the medium being changed after 48 hours to one that contained either 15% (v/v) $^{63}Ni^{2+}$ labelled serum or 15% (v/v) normal serum together with sufficient $^{63}Ni^{2+}$ muscle diffusate to give a final concentration of 7–10 $\mu$g $^{63}Ni^{2+}$/ml. After a further 48 hours the medium was removed, the cell sheets being washed 4 times with fresh, unlabelled medium, and then treated with a solution (2·0 ml) of either 1% (w/v) Difco 1 : 250 trypsin (Difco Laboratories Inc., Detroit, USA) or crystalline trypsin (Sigma 2X crystallized 500 $\mu$g/ml) in $Ca^{2+}$ and $Mg^{2+}$ free, Tyrode solution supplemented with 1% (w/v) sodium citrate (Rinaldini, 1959). After 10 minutes at 37° C an equal volume of 15% (v/v) serum in Waymouth's medium was added to each bottle to inhibit the trypsin and the cells were dislodged by gentle agitation. In some experiments the cells were liberated by treatment with collagenase (Sigma, Type 1; 1 mg/ml) instead of trypsin. The cells were recovered from the combined suspensions by centrifugation (200*g*; 5 minutes) washed 4 times in 0·9% (w/v) NaCl and finally made up to a convenient volume (10 or 12 ml) in saline. Samples of this cell suspension were taken for haemocytometer cell counts, $^{63}Ni^{2+}$ assay, DNA analysis and cell fractionation. In certain of these experiments the cells from one culture bottle, selected at random, were harvested with sterile precautions and subcultured in fresh unlabelled medium, either in bottles to check viability, or on 3 × 1 in slides in Petri dishes for subsequent autoradiography.

*Autoradiography.*—The slide cultures were incubated for 24–48 hours in McIntosh jars in an atmosphere of $N_2$ (75%), $O_2$ (20%) and $CO_2$ (5%), then washed in 0·9% (w/v) NaCl, fixed in absolute methanol and coated with Ilford K5 Nuclear Research emulsion (gel form) at 40° C (Messier and Leblond, 1957). The slides were processed after 1, 4 and 5 weeks, the underlying cells being stained with Meyer's carmalum (Romeis, 1932).

*Cell fractionation.*—The cells were separated from the saline medium by centrifugation, resuspended in medium RSB (10 mmol/l NaCl, 1·5 mmol/l KCl and 10 mmol/l tris HCl buffer, pH 7·4; 10 ml, Penman, 1966) and fractionated as described by Zimmerman *et al.* (1969). The process of cell breakage during the initial homogenization was followed microscopically and was continued until the number of intact cells per ml was reduced by at least 80%. The isolated subcellular particulate fractions (Fig. 1) were resuspended in water or RSB for analysis. Nucleolar preparations were examined microscopically, methanol-fixed smears being stained by the methods given previously (Webb, Heath and Hopkins, 1972).

*Determination of radioactivity.*—Samples for analysis were digested with 1 ml Aristar $HNO_3$ (BDH Ltd.), the digests being transferred quantitatively with water to scintillator vials and then evaporated *in vacuo* over $P_2O_5$ and KOH. When completely dry, the residues were dissolved in formic acid (0·5 ml)

FIG. 1.—Experimental procedure for the fractionation of cultured mouse dermal fibroblasts (strain C57S/IP).

and the solutions warmed to 60–70° C until the decomposition of any residual nitrate was complete. After the addition of scintillant (10 ml of a solution of 2,5-diphenyloxazole (4·0 g) and 1,4-bis-(5-phenyloxazol-2-yl)-benzene (0·1 g) in a mixture of toluene (700 ml) and 2-ethoxyethanol (300 ml), Hall and Cocking, 1965) radioactivity was measured with an efficiency of 66% in a Packard Model 3375 Tricarb Liquid Scintillation Spectrometer (50–750 window; 16% grain). (The organic scintillants were obtained from Koch-Light Laboratories Ltd., Colnbrook.) Quench corrections were obtained by recounting the samples after the addition of an aqueous solution (50 $\mu$l) of $^{63}NiCl_2$ (0·01 $\mu$Ci/ml) as an internal standard.

*Analytical methods.*—Ribonucleic acid and DNA were determined by the methods of Mejbaum (1939) and Burton (1956), respectively.

### RESULTS

When cultures of the C57S/IP strain of mouse fibroblasts were grown in the $^{63}Ni^{2+}$ labelled serum medium, $^{63}Ni^{2+}$ was taken up by the monolayer of cells but binding of the cation by the intercellular material was much greater than the intracellular incorporation. In culture 3 (Table I), for example, the cation uptake by the monolayer after 48 hours, as measured by the decrease in $^{63}Ni^{2+}$ content of the medium, was 3·5% (0·72 $\mu$g ions) of that available in solution. After trypsinization, however, only 0·023 $\mu$g ions $^{63}Ni^{2+}$ were recovered in the isolated cells, whereas almost 0·7 $\mu$g ions were present

TABLE I.—*Intracellular Distribution of $^{63}Ni^{2+}$ in Mouse Dermal Fibroblasts (Strain C57S/IP) after Growth in $^{63}Ni^{2+}$ Serum and $^{63}Ni^{2+}$ Muscle Diffusate*

| Culture No. | Addition to growth medium | Concentration of $^{63}Ni^{2+}$ in growth medium µmol/ml* | $^{63}Ni^{2+}$-incorporated nmol/10⁶ cells | Intracellular distribution of $^{63}Ni^{2+}$ (% of $^{63}Ni^{2+}$ recovered in all fractions): Mito-chondrial fraction | Micro-somal fraction | Cell sap fraction | Nuclear fraction | Intranuclear distribution of nuclear $^{63}Ni^{2+}$ (% of $^{63}Ni^{2+}$ in nuclei): Nucleoli | Nuclear sap+DNA |
|---|---|---|---|---|---|---|---|---|---|
| 1 | $^{63}Ni^{2+}$ serum | 0·173 | 0·53 | 19·0 | 10·6 | 34·3 | 36·1 | 45·2 | 16·6 |
| 2 | $^{63}Ni^{2+}$ serum | 0·173 | 0·16 | 20·9 | 7·9 | 35·2 | 36·0 | 43·5‡ | 10·2 |
| 3 | $^{63}Ni^{2+}$ serum | 0·194 | 0·30 | 24·6 | 10·2 | 27·4 | 37·8 | 47·2 | 29·8 |
| 6 | $^{63}Ni^{2+}$ serum | 0·194 | 0·36 | 26·1 | 12·7 | 21·8 | 39·4 | — | — |
| 4 | $^{63}Ni^{2+}$ muscle diffusate | 0·113 | 0·38 | 21·6 | 12·7 | 29·5 | 36·2 | 31·3 | — |
| 5 | $^{63}Ni^{2+}$ muscle diffusate | 0·159† | 0·24 | 24·2 | 9·9 | 32·2 | 33·7 | 68·6 | 21·8 |

* 1 µmol $^{63}Ni^{2+}$ had $2 \cdot 01 \times 10^5$ counts/minute.

† In these cultures the medium was changed after 24 hours.

‡ 3·9 nmol $Ni^{2+}$ /mg RNA.

The cultures were grown and fractionated as described in the " Material and Methods " Section.

FIG. 2.—Uptake of $^{63}Ni^{2+}$ from $^{63}Ni^{2+}$ labelled serum by stationary phase C57S/IP cells. Monolayer cultures of mouse dermal fibroblasts (strain C57S/IP) were grown for 72 hours in normal medium and then transferred to Waymouth's medium containing 15% (v/w) $^{63}Ni^{2+}$ serum ($\equiv$ 12·2 $\mu$g $^{63}Ni^{2+}$/ml medium). The cells were harvested at different times after transfer, counted and analysed for $^{63}Ni^{2+}$ as described in the " Materials and Methods " section. The number of cells per culture bottle was 4·6 $\times$ $10^6$ ($\pm$37%).

in the soluble products of the enzyme digestion. Because of this binding of $^{63}Ni^{2+}$ by the intercellular matrix, which suggests that the serum-bound cation is freely exchangeable (see also Weinzierl and Webb, 1972), the location of the intracellularly incorporated isotope was not clearly defined in autoradiographs of monolayer slide cultures. Such preparations, for example, showed silver granules randomly distributed between the cells but no consistent intracellular location except in an occasional, intensely stained pyknotic nucleus, presumably in a dead cell, where silver grains were conspicuous, both intranuclearly and concentrated at the nuclear membrane.

Incorporation of $^{63}Ni^{2+}$ from $^{63}Ni^{2+}$ labelled serum (12·2 $\mu$g $Ni^{2+}$/ml medium) by stationary phase C57S/IP cells in culture was linear for about 30 hours, and was essentially complete after 48 hours (Fig. 2). Thus, as shown by the results of Fig. 2, the cellular content of the cation increased by only a further 5% between 48 and 72 hours. Viability was not affected significantly by this concentration of $Ni^{2+}$ in the medium, since cells from such cultures grew as well as those from untreated controls when transferred to fresh $Ni^{2+}$ free medium.

After growth of cells for 48 hours in media that contained $^{63}Ni^{2+}$ labelled serum or $^{63}Ni^{2+}$ muscle diffusate, a common pattern was observed in the intracellular distribution of the cation (Table I). Thus about 60–70 % of the incorporated $^{63}Ni^{2+}$ was associated with the nuclear and cell-sap fractions, the content in the former being similar to, but greater than, that in the latter; 20–25 % in the mitochondrial fraction and 10 % in the microsomal fraction. These results (Table I) have been corrected to allow for the 10–20 % of the cells that remained unbroken after homogenization, and which sedimented with the nuclear fraction on centrifugation (Fig. 1), but otherwise a quantitative recovery of all particulate components has been assumed. In terms of radioactivity, however, an average loss of 10 % of the total counts occurred during fractionation of the labelled cells, whereas only about 85 % of the cellular DNA was recovered in the isolated nuclear fraction. It is probable, therefore, that the values given in Table I for the contents of $^{63}Ni^{2+}$ in the nuclear fractions are low. Also, it is possible that some redistribution of the cation may have occurred during the fractionation procedure (Fig. 1). It is unlikely, for example, that the mitochondria and microsomes would remain undamaged in the hypotonic RSB medium although nuclei and nucleoli isolated from HeLa cells by this method are active in protein synthesis (Zimmerman *et al.*, 1969).

About 50 % of the $^{63}Ni^{2+}$ bound by the nuclei of the C57S/IP cells was recovered in the nucleoli (Table I). By microscopy, preparations of these organelles were consistently good, the levels of contamination by other particles and whole nuclei being extremely small. Since in the present work the nucleoli were isolated after treatment with DNase (Fig. 1), the average RNA : DNA ratio (1·5 : 1) differed from that of the nucleolar preparations from primary nickel-induced rhabdomyosarcomata (RNA : DNA 0·33 : 1; Webb *et al.*, 1972). Also, in the fractionation of the C57S/IP nuclei, only about 60 % of the nuclear $^{63}Ni^{2+}$ was recovered in the nucleolar and "nuclear sap + DNA" fractions, whilst 30–40 % was removed in the supernatant fraction, S4 (Fig. 1) after the preliminary detergent treatment of the nuclei. This loss was too great to be due to the removal of residual cytoplasm and unbroken cells from the nuclear preparations which, according to Penman (1966) is the function of the detergent mixture. It seems probable that the latter affected both the permeability of the nuclear membrane and the solubility of the chromatin since, as observed previously by Zimmerman *et al.* (1969), this treatment caused the nuclei to gel.

### DISCUSSION

The present results show that $^{63}Ni^{2+}$, when added to the culture medium in the form of complexes with either protein or the small, diffusible molecules from a sterile autolysate of rat muscle, is incorporated intracellularly by mouse dermal fibroblasts (strain C57S/IP) *in vitro*. Although quantitative measurements of the intracellular distribution of the incorporated $^{63}Ni^{2+}$ are subject to certain limitations, as discussed above, it seems probable that the cation, irrespective of the nature of the carrier molecules, is bound in decreasing order of amount by the nuclei, cell sap, mitochondria and microsomes. The same order has been found for the intracellular distribution of $Ni^{2+}$, $Co^{2+}$ and $Cd^{2+}$ ions in rhabdomyosarcomata induced by implants of metallic nickel, cobalt and cadmium in the rat although in these primary tumours binding of the appropriate cation in the cell nucleus is much greater than in any other fraction, *i.e.* about 80–90 % of the cellular content (Heath and Webb, 1967).* In the nuclei from these tumours,

* In the *in vitro* system, slight contamination of the cells by $^{63}Ni^{2+}$, present in intercellular material and released on treatment of the monolayer cultures with trypsin, could lead to erroneously high values for the cation content of the cell sap fraction.

53 % (range 41–63 %) of the nuclear $Ni^{2+}$ is associated with the nucleoli (Webb *et al.*, 1972); the corresponding value for the nuclei of the C57S/IP cells is 47 % (range 31–69 %, Table I). This close parallel between the intranuclear location of $Ni^{2+}$ in the primary nickel-induced tumours and in cells cultured for short periods in the presence of $Ni^{2+}$ complexes seems significant and may indicate that one effect common to the action of the carcinogenic metal *in vivo* and *in vitro* may be interference with nucleolar function.

The authors are grateful to Miss D. Jackson and Mr G. Payton for technical assistance with this work.

### REFERENCES

BURTON, K. (1956) A Study of the Conditions and Mechanisms of the Diphenylamine Reaction for the Colorimetric Estimation of Deoxyribonucleic Acid. *Biochem. J.*, **62**, 315.

DANIEL, M. R. (1969) The Effect of Malignant Dermal Cells on Embryonic Epidermis *in vitro*. *Br. J. Cancer*, **23**, 861.

HALL, T. C. & COCKING, E. C. (1965) High Efficiency Liquid-scintillation Counting of $^{14}C$-labelled materials in Aqueous Solution and Determination of Specific Activity of Labelled Proteins. *Biochem. J.*, **96**, 626.

HEATH, J. C. & WEBB, M. (1967) Content and Intracellular Distribution of the Inducing Metal in the Primary Rhabdomyosarcomata Induced in the Rat by Cobalt, Nickel and Cadmium. *Br. J. Cancer*, **21**, 768.

HEATH, J. C., WEBB, M. & CAFFREY, M. (1969) Interaction of Carcinogenic Metals with Tissue and Body Fluids, Cobalt and Horse Serum. *Br. J. Cancer*, **23**, 153.

MEJBAUM, W. (1939) Estimation of Small Amounts of Pentose, Especially in Derivatives of Adenylic Acid. *Z. physiol. Chem.*, **258**, 117.

MESSIER, B. & LEBLOND, C. P. (1957) Preparation of Coated Radioautographs by Dipping Sections in Fluid Photographic Emulsion. *Proc. Soc. exp. Biol. Med.*, **96**, 7.

PENMAN, S. (1966) RNA Metabolism in the HeLa Cell Nucleus. *J. molec. Biol.*, **17**, 117.

RINALDINI, L. M. (1959) An Improved Method for the Isolation and Quantitative Cultivation of Embryonic Cells. *Expl Cell Res.*, **16**, 477.

ROMEIS, B. (1932) *Taschenbuch der Mikroscopischen Technik* (13th Ed.). München and Berlin: R. Oldenbourg.

WAYMOUTH, C. (1959) Rapid Proliferation of Sublines of NCTC Clone 929 (Strain L) Mouse Cells in a Simple Chemically Defined Medium (MB 752/1). *J. natn. Cancer Inst.*, **22**, 1003.

WEBB, M., HEATH, J. C. & HOPKINS, T. (1972) Intranuclear Distribution of the Inducing Metal in Primary Rhabdomyosarcomata Induced in the Rat by Nickel, Cobalt and Cadmium. *Br. J. Cancer*, **26**, 274.

WEINZIERL, S. M. & WEBB, M. (1972) Interactions of Carcinogenic Metals with Tissue and Body Fluids. *Br. J. Cancer*, **26**, 279.

ZIMMERMAN, E. F., HACKNEY, J., NELSON, P. & ARIAS, I. M. (1969) Protein Synthesis in Isolated Nuclei and Nucleoli of HeLa Cells. *Biochemistry*, **8**, 2636.